# Processing of Tuna Head By-Products into Antioxidant Peptide Ingredients for Aquaculture Feeds

**DOI:** 10.3390/antiox14070770

**Published:** 2025-06-23

**Authors:** Raúl Pérez-Gálvez, F. Javier Espejo-Carpio, Pedro J. García-Moreno, Antonio Guadix, Emilia M. Guadix

**Affiliations:** Department of Chemical Engineering, Faculty of Sciences, University of Granada, 18071 Granada, Spain; fjespejo@ugr.es (F.J.E.-C.); pjgarcia@ugr.es (P.J.G.-M.); aguadix@ugr.es (A.G.)

**Keywords:** skipjack tuna (*Katsuwonus pelamis*) heads, fish farming, enzymatic hydrolysis, membrane separation, radical scavenging activity, metal chelation

## Abstract

This study aimed to produce antioxidant peptide fractions from Skipjack tuna (*Katsuwonus pelamis*) head by-products through enzymatic hydrolysis and membrane filtration. Raw materials were sequentially hydrolyzed with Alcalase^®^ (4 h) and Flavourzyme^®^ (1 h), reaching a final degree of hydrolysis of 18.5 ± 0.9%. The crude hydrolysate was fractionated using ceramic membranes with molecular weight cut-offs of 8, 3, and 1 kDa. Some peptide fractions presented a relevant proportion of short-chain peptides (>50% *w*/*w*) and free amino acids (>10% *w*/*w*), as well as a high content of essential amino acids (>64% mol), supporting their value as dietary ingredients for aquafeeds. In vitro antioxidant activities were assessed by 2,2-diphenyl-1-picrylhydrazyl (DPPH) radical scavenging and ferrous ion chelation assays. Some fractions (e.g., F3R1 with IC_50_ = 1.04 ± 0.01 mg·mL^−1^ for metal chelating activity) displayed significantly improved (*p* < 0.05) antioxidant properties compared to the unfractionated hydrolysate (IC_50_ = 2.75 ± 0.08 mg·mL^−1^). This may be linked to their molecular weight profile and hydrophobic amino acid content. These results demonstrate the potential of the proposed approach to obtain bioactive peptide fractions with functional properties for aquafeeds. Zootechnical trials are needed to assess their effects on feed utilization and in vivo mitigation of oxidative stress.

## 1. Introduction

According to the State of World Fisheries and Aquaculture 2024 (SOFIA), worldwide production of aquatic animals from capture fisheries and aquaculture reached a record of 185 million tonnes in 2022, where aquaculture farming represented 51% of total production, surpassing extractive catches. This report states that 89% of total aquatic production was destined for human consumption, estimating an average consumption of 20.7 kg per capita [1]. The increase in fish and seafood consumption is mostly associated with higher demand for processed products rather than raw fish. Fish and seafood processing involves several operations (e.g., heading, evisceration, boiling, and marinating), which generates large quantities of by-products (e.g., fillet remains, heads, viscera, bones, scales, and fins) which represent 55–65% in weight of the global catch per year [2].

The tuna canning industry plays a relevant role in the Spanish fish processing sector, representing more than 70% of the total value of canned products produced in 2024 [3]. The main by-products resulting from tuna canning operations include heads (13% *w*/*w*), skin (10% *w*/*w*), viscera (8% *w*/*w*), bones (6% *w*/*w*), and fins (1% *w*/*w*) [4]. Their specific protein and lipid composition make tuna by-products suitable for processing into fish oil and protein hydrolysates. For instance, tuna heads present on average 15% *w*/*w* proteins and 13.5% *w*/*w* lipids, as well as being a source of minerals such as hydroxyapatite or calcium phosphates [5,6,7]. The enzymatic valorization of tuna by-products into protein hydrolysates represents a promising strategy to enhance the economic value of these underutilized materials. By-products from tuna species such as skipjack (*Katsuwonus pelamis*) and yellowfin tuna (*Thunnus albacares*) have been widely reported as substrates for producing enzymatic hydrolysates containing bioactive peptides. These peptides, which remain inactive within the native protein structure, exert biological activity upon release through enzymatic hydrolysis. Among their bioactivities, antioxidant effects are the most extensively studied due to their relevance in health promotion and disease prevention. For example, hydrolysates derived from skipjack tuna milt or gelatin have demonstrated strong in vitro radical scavenging and metal chelation capacities. After purification via ultrafiltration membranes and chromatographic methods, several isolated peptides have shown in vivo protective effects against oxidative cell damage induced by H_2_O_2_ or skin injury caused by ultraviolet-A radiation [8,9]. Sisa et al. [10] valorized yellowfin tuna tails to obtain different added-value products such as muscle proteins—evaluated as a nitrogen source for protease growth—and gelatins from tuna skins. These were further hydrolyzed by an optimized enzymatic procedure to release antioxidant peptides with maximal DPPH radical scavenging activity. The antioxidant capacity of papain hydrolysates from yellowfin tuna skin collagen papain has been confirmed by in silico (i.e., molecular docking) and in vitro (i.e., DPPH radical scavenging activity) assays [11]. Beyond their antioxidant properties, peptides derived from tuna by-products exhibit diverse biological activities, including Angiotensin I-converting enzyme (ACE) inhibitory activity [12]—linked to blood pressure regulation—as well as antimicrobial [13] or immunomodulatory [14] capacities. These studies support the use of these underutilized biomaterials in the food and pharmaceutical industries.

Fish processing by-products can also be valorized as sustainable protein sources for aquafeed production. In contrast to seafood, fish farming depends on the use of compound feeds, with national consumption in Spain reaching 181,000 tonnes in 2023 [15]. The formulation of aquafeeds (i.e., selection of the raw material, inclusion, and evaluation of the digestibility) is crucial to ensure the viability and sustainability of aquaculture systems. In this sense, protein represents a critical component in feed formulation, with dietary inclusion levels recommended at 50–65% by weight for larval stages [16], decreasing progressively for juvenile and adult stages. Fishmeal remains the predominant protein source in aquafeeds, with approximately 86% of its global production intended for aquafeed manufacture [1].

The sharp increase in demand for fish meal for aquaculture and livestock farming, combined with declining fishery catches, has doubled the cost of this ingredient over the past two decades, driving the search for alternative protein sources [17]. In this context, by-products from the fish industry represent a cost-effective and renewable feed source that can meet the nutritional requirements for protein and lipids of aquaculture species [18]. However, their direct use in aquafeeds may be technically limited by the presence of insoluble components and their deficient digestibility in larvae and juveniles [19,20]. In this regard, several studies suggest the partial replacement of crude fish protein by enzymatic hydrolysates, improving the digestibility and palatability of aquafeeds [21,22,23,24].

Besides their improved digestibility, some marine peptides can display specific biological activities, such as antimicrobial, anti-inflammatory, immunomodulatory, or antioxidant, among others [25,26,27]. The incorporation of natural antioxidants, such as specific peptides, into aquafeeds serves two main purposes: (i) extending the shelf life of the feed by limiting the oxidation of lipid ingredients (e.g., triglycerides, phospholipids, and lecithin) formulated alongside proteins; (ii) mitigating the oxidative stress in farmed species. The latter is achieved by neutralizing the reactive oxygen species (ROS) generated by environmental factors commonly present in aquaculture systems (e.g., high rearing densities, presence of ammonia, heavy metals, pathogens, or other stressors) [21,28].

Fish protein hydrolysates are complex mixtures comprising undigested proteins, peptides, and free amino acids. To obtain the bioactive peptides, it is essential to separate the raw hydrolysate into fractions based on molecular weight. In this context, the combination of enzymatic hydrolysis with membrane separation is extensively reported in the literature as an effective strategy for the simultaneous production and recovery of peptide fractions with improved biological activity [13,29]. Membrane separation performance is often limited by fouling, the irreversible accumulation of feed components on the membrane surface or within its pores. Therefore, an efficient membrane fractionation process requires the appropriate selection of membrane characteristics (e.g., material and molecular weight cut-off) and operating parameters (e.g., crossflow velocity and transmembrane pressure), as well as effective strategies for fouling control and membrane cleaning [30].

This study proposes an integrated process for obtaining antioxidant peptide fractions from tuna head by-products, intended as functional ingredients for aquaculture feeds. To this end, tuna heads from the canning industry were enzymatically hydrolyzed using sequential treatment with Alcalase and Flavourzyme. The fish protein hydrolysates, rich in short-chain peptides and free amino acids, were then fractionated via sequential ultrafiltration using ceramic membranes with 1, 3, and 8 kDa molecular weight cut-offs, yielding peptide fractions with narrow molecular weight distributions. These fractions were evaluated for their in vitro antioxidant activity, including DPPH radical scavenging and ferrous ion chelation. This procedure is suitable for recovering peptide fractions with antioxidant activity to take part as functional ingredients of aquafeeds.

## 2. Materials and Methods

### 2.1. Production of Tuna Head Hydrolysates

Skipjack tuna (*Katsuwonus pelamis*) heads were obtained from the fish canning factory Conservera de Tarifa (Tarifa, Cádiz, Spain). These materials were ground and kept refrigerated at −20 °C prior to analysis. The average proximate composition of tuna head, determined according to the official methods [31], was 38.4% protein, 9.6% lipid, and 11.5% mineral matter.

Tuna heads were ground and homogenized with water, obtaining 2 L of suspension with 15% *w*/*w* of protein. The protein suspension was transferred to a stirred tank reactor, immersed in a water bath to maintain a constant reaction temperature. The reaction mixture was then subjected to a 5 h enzymatic treatment at pH 7.5 and 55 °C. The reaction started by the addition of Alcalase 2.4 L^®^ (Novozymes, Bagsværd, Denmark) at an enzyme/substrate ratio of 3% *w*/*w*. After completing 4 h, the enzyme preparation Flavourzyme^®^ (Novozymes, Denmark) was added to the reaction mixture at an enzyme/substrate ratio of 3% *w*/*w*. The reaction was then allowed to proceed for 1 h. These operation conditions are based on previous research on tuna heads, where the combined enzymatic treatment was optimized to produce a final hydrolysate enriched in short-chain peptides and free amino acids [32].

The pH was monitored and maintained throughout the reaction using an automated titrator (718 Stat Titrino, Metrohm, Switzerland). According to the pH-stat method [33], the degree of hydrolysis (DH), defined as the number of available peptide bonds cleaved by proteolysis, was related to the amount of titration agent (NaOH 1 N) added to keep a constant pH throughout the reaction.

(1) $$\mathrm{DH}\%=\frac{V_{b}\cdot N_{b}}{\alpha \cdot m_{P}\cdot h_{\mathrm{tot}}}\times 100$$

where V_b_ and N_b_ stand for the volume (mL) and normality (meq·mL^−1^) of base consumed, m_P_ (g) is the mass of protein fed to the reactor, h_tot_ is the number of milliequivalents of peptide bonds per gram of protein (8.6 meq·g^−1^ of protein), and α is the average degree of dissociation of the α-amino group, which was assumed to be 0.7320 for pH 7.5 and 55 °C [33,34].

The enzymatic reaction was stopped by heating at 100 °C for 15 min, assuring enzyme thermal inactivation. The resulting tuna head hydrolysate (THH) was cooled down to room temperature and filtered through a Büchner funnel with Whatman cellulose paper of 8 µm pore size to remove insoluble materials (e.g., scales and fishbones) and fats. The prefiltered hydrolysate was kept at 4 °C for 15 h at the most prior to membrane fractionation.

### 2.2. Fractionation of the Tuna Head Hydrolysates with Ceramic Ultrafiltration Membranes

#### 2.2.1. Operation of the Ceramic UF Membranes

The prefiltered hydrolysates were separated into different peptide fractions by a sequential ultrafiltration treatment. Three ceramic ultrafiltration membranes (Tami, Nyons, France) of molecular weight cut-offs (MWCOs) of 8, 3, and 1 kDa were selected for the fractionation. The membranes consisted of a single tubular channel with a hydraulic diameter of 6 mm and length of 25 cm, providing a filtration area of 0.0042 m^2^. The ultrafiltration (UF) modules were operated in batch concentration mode, yielding a permeate stream, while the retentate was recycled to the feed vessel. For each batch concentration, 2 L of feed solution was filtered at 50 °C, 1 bar of transmembrane pressure, and a crossflow velocity of 3.3 m·s^−1^. This operation proceeded until attaining a volume concentration factor of 1.6. This parameter was defined as the ratio between the volume of permeate collected and the initial volume (2 L) of the feed solution. The time required for the concentration varied from 2.5 h to 4.8 h, depending on the membrane MWCO.

**Figure 1:**
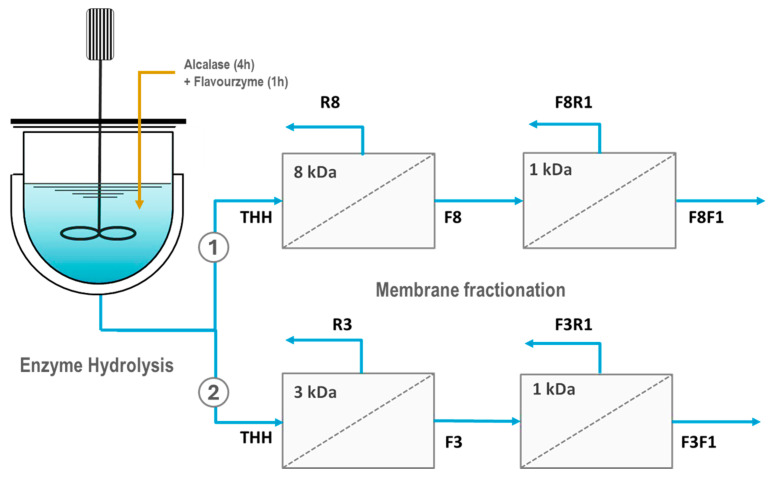
Process flowsheet showing the enzyme reactor and the two membrane configurations employed to obtain bioactive peptide fractions from Skipjack tuna by-products. THHs: Tuna Head Hydrolysates. R8, R3: retentate streams from the 8 and 3 kDa MWCO membranes, respectively. F8, F3: permeate streams from the 8 and 3 kDa MWCO membranes, respectively. F8R1, F3R1: retentate streams obtained after filtration through 1 kDa of F8 and F3, respectively. F8F1, F3F1: permeate streams obtained after filtration through 1 kDa of F8 and F3, respectively.

The ceramic ultrafiltration membranes were arranged into two different configurations, each one comprising two consecutive UF units of decreasing molecular weight cut-off (8 + 1 kDa; 3 + 1 kDa). As shown in Figure 1, both sequences allowed the recovery of several fractions, noted as R (i.e., retentate streams) and F (i.e., filtrate streams). For instance, the THH feed filtered through the 8 kDa membrane was separated into the fractions R8 and F8. The permeate F8 was subsequently filtered through the 1 kDa membrane, yielding the retentate fraction F8R1 (i.e., the fraction passing through the 8 kDa membrane but retained by the 1 kDa membrane) and the permeate F8F1 (i.e., the peptide fraction permeating through both membranes). Similarly, the membrane sequence 3 + 1 kDa separated the hydrolysate into the peptide fractions R3, F3R1, and F3F1. Each membrane fraction was freeze-dried and kept at −20 °C before analysis.

#### 2.2.2. Determination of Membrane Permeability

Before operation, each ceramic membrane was hydrated with de-ionized water at 50 °C for 1 h, with recirculation of both permeate and retentate streams to the feed tank. At this point, the water permeability of the membrane was determined by passing de-ionized water at 50 °C and a crossflow velocity of 3.3 m·s^−1^, measuring the permeate flux (i.e., volumetric flow of permeate per unit of membrane area) at increasing transmembrane pressure. The water permeability of the three ceramic membranes (8, 3, and 1 kDa) was evaluated by linear regression of the observed values of water flux (J_w_) against the applied transmembrane pressure (TMP). The membrane intrinsic resistance (R_M_) was calculated as the inverse of the slope of water permeate flux against the transmembrane pressure according to Equation (2).

(2) $$R_{M}=\frac{\mathrm{TMP}}{J_{w}}$$

#### 2.2.3. Modeling of the Permeate Flux

The permeate flux was fitted to a dynamic model based on the series-resistance approach [35,36,37]. This classic model considers that the total resistance against membrane filtration can be split into three contributions: the resistance provided by the membrane active layer (R_M_), the resistance of the concentration polarization layer (R_CP_), and the resistance due to the irreversible deposition of fouling materials (R_F_). The fouling resistance is time-dependent, as it increases with the deposition of foulant solutes on the membrane surface, which is assumed to follow first-order kinetics with rate constant k.

(3) $$J=\frac{\mathrm{TMP}}{R_{M}+R_{\mathrm{CP}}+R_{F}\left(t\right)}$$

In crossflow ultrafiltration, the permeate flux does not decrease indefinitely. Still, it reaches a minimum steady value J_∞_. At this point, the tangential flow counteracts the deposition of new solutes on the membrane surface, preventing fouling. All these assumptions lead to Equation (4):

(4) $$J=\frac{J_{0}}{1+\left(\frac{J_{0}-J_{\infty }}{J_{\infty }}\right)\cdot \left(1-e^{-k\cdot t}\right)}$$

where J_0_ and J_ꝏ_ are the initial and steady values of observed permeate flux, respectively. The kinetic constant k represents the rate of deposition of foulant materials onto the membrane surface.

#### 2.2.4. Cleaning Treatment of the Ceramic UF Membranes

An alkaline cleaning treatment was proposed to restore the permeability of the membranes after operation. The cleaning sequence started with an initial rinse of the permeate port with demineralized water at 3.3 m·s^−1^ and no transmembrane pressure. Then an alkali solution, containing 20 g·L^−1^ NaOH plus 2 g·L^−1^ Sodium Dodecyl Sulphate (SDS), was passed through the membrane at 50 °C and a crossflow velocity of 3.3 m·s^−1^ for 30 min. The cleaning protocol was concluded with a final neutralization step with demineralized water until attaining neutral pH in both permeate and retentate streams. The percentage efficiency of the cleaning treatment (%E) was then calculated by Equation (5):

(5) $$\%E=\frac{R_{1}-R_{\mathrm{NaOH}}}{R_{1}-R_{M}}\times 100$$

where R_M_ is the intrinsic membrane resistance, evaluated before operation by Equation (2), R_1_ is the resistance of the fouled membrane after UF, and R_NaOH_ is the hydraulic resistance provided by the ceramic membrane after the alkaline cleaning treatment.

### 2.3. Characterization of the Membrane Peptide Fractions

#### 2.3.1. Determination of Soluble Protein of the Peptide Fractions

The concentration of soluble protein in the crude hydrolysate (THH) and the permeate and retentate membrane fractions was determined using the Bicinchonicic Acid (BCA) method [38]. This procedure is based on the reduction of cupric ions by proteins under alkaline conditions. The reduced Cu(I) forms a complex with BCA, which can be detected spectrophotometrically at 562 nm.

#### 2.3.2. Amino Acid Composition of the Fractions F3F1 and F3R1

The amino acid composition of the membrane fractions F3F1 and F3R1, permeate and retentate streams obtained after successive filtration of the crude hydrolysate through 3 and 1 kDa, was determined using an automatic amino acid analyzer Biochrom 30 with ion exchange chromatography (Biochrom, Cambridge, UK). The samples were digested with 6 M HCl at 110 °C for 20 h, prior to analysis. The digested samples were then cooled and dissolved in sodium citrate buffer (pH 2.2). The amino acid profile was determined through ninhydrin color reaction and photometric detection at 570 nm. The results were expressed as molar percentage.

#### 2.3.3. Molecular Weight Distribution of the Peptide Fractions

The molecular weight distribution of the UF fractions was determined by size exclusion chromatography (SEC) on a Superdex peptide 100/300 GL column (GE, Health care, Uppsala, Sweden). To this end, freeze-dried fractions were dissolved in ultrapure water with 10 mg of protein·mL^−1^. A volume of 100 μL was injected to the SEC column eluted at 0.5 mL·mL^−1^, employing as the mobile phase a mixture 70:30 of ultrapure water with acetonitrile. The molecular weight composition of the UF fractions was reported as the percentage area of the absorbance at 280 nm against the retention time. A calibration curve was obtained by employing as standards glycine (Gly, 75 Da), alanine (Ala, 89 Da), tripeptide Phe-Gly-Gly (279 Da), hexapeptide (Gly)6 (360 Da), vitamin B12 (1355 Da), insulin (5733 Da), aprotinin (6511 Da), and ribonuclease (13,700 Da).

### 2.4. Determination of the Antioxidant Activities of the Peptide Fractions

Two antioxidant properties were investigated in the UF fractions, namely the ability to sequestrate DPPH radicals and ferrous ion chelating activity. The scavenging activity of 2,2-diphenyl-1-picrylhydrazyl (DPPH) radicals was determined by the method of Picot et al. [39]. To this end, a serial dilution of hydrolysates (1 mL) was mixed with 1 mL of 0.1 mM DPPH solution in methanol. After incubation for 30 min, the absorbance was measured at 515 nm. DPPH radical scavenging activity was calculated by Equation (6):

(6) $$\mathrm{DPPH}\;\mathrm{radical}\;\mathrm{scavenging},\;\%=\left(1-\frac{A_{\mathrm{sample}}-A_{\mathrm{control}}}{A_{\mathrm{blank}}}\right)$$

where the control sample was obtained by replacing DPPH radical solution with methanol, and the blank was a 1:1 mixture of DPPH radical solution and distilled water.

Ferrous ion chelating capacity was determined following the method proposed by Decker and Welch [40]. A set of hydrolysate dilutions (1 mL) was mixed with 3.7 mL of distilled water and 0.1 mL of FeCl_2_ solution. After incubation for 3 min at room temperature, a volume of 0.2 mL of Ferrozine^TM^ 5 mM was added and the new solution was incubated for 10 min. The absorbance was then measured at 562 nm. Percentage ferrous ion chelating activity was calculated by Equation (7):

(7) $${\mathrm{Fe}}^{2+}\;\mathrm{chelating},\;\%=\left(1-\frac{A_{\mathrm{sample}}-A_{\mathrm{control}}}{A_{\mathrm{blank}}}\right)$$

where the control sample was prepared without the addition of Ferrozine^TM^ and the blank solution contained water instead of hydrolysate solution. Both antioxidant properties were reported as the halfway inhibitory concentration (IC_50_, mg/mL).

### 2.5. Statistical Analysis

Data were subjected to analysis of variance (ANOVA) by using Statgraphics Centurion XVI ™ (Statistical Graphics Corp., Rockville, MD, USA). Tukey’s multiple range test was used to evaluate significant differences between the molecular weight distribution and in vitro antioxidant activities of the peptide fractions. Differences between mean values were considered significant at a level of confidence of 95% (*p* < 0.05).

## 3. Results and Discussion

### 3.1. Production of the Tuna Head Hydrolysates

**Figure 2:**
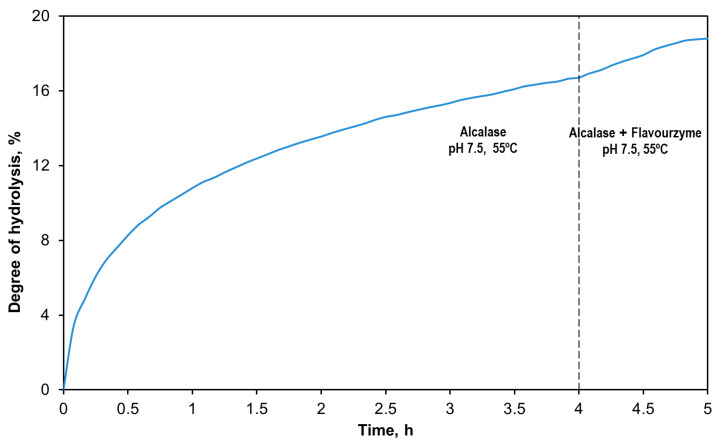
Time evolution of the percentage DH for the enzymatic treatment with Alcalase (4 h, pH 7.5, 55 °C) + Flavourzyme (1 h, pH 7.5, 55 °C).

Figure 2 illustrates the evolution of DH over the 5 h reaction. The hydrolysis curve can be divided into two regions, corresponding to the sequential enzymatic treatment. During the initial Alcalase stage, DH increased rapidly, followed by a gradual plateau as the concentration of accessible peptide bonds decreased. The addition of Flavourzyme after 4 h resulted in an immediate increase in DH; however, it soon stabilized around DH 18.5 ± 0.9%.

Alcalase is a commercial preparation derived from *Bacillus licheniformis*, with subtilisin as its main enzymatic component. Subtilisin is a broad-spectrum serine endoprotease that preferentially cleaves peptide bonds with hydrophobic and aromatic residues. It exhibits maximal proteolytic activity at alkaline pH (8–9) and at temperatures ranging from 50 to 60 °C [33,41]. Due to its broad specificity and high proteolytic activity within the experimental conditions (pH 7.5 and 55 °C), it is expected that the extent of hydrolysis is primarily determined by the Alcalase treatment. In contrast, Flavourzyme is a commercial enzymatic cocktail, derived from *Aspergillus oryzae,* mostly composed of amino- and dipeptidases. According to Merz et al. [42], Flavourzyme shows optimal peptidase activity at near-neutral pH (6–7) and temperatures in the range 55–65 °C. 

**Figure 3:**
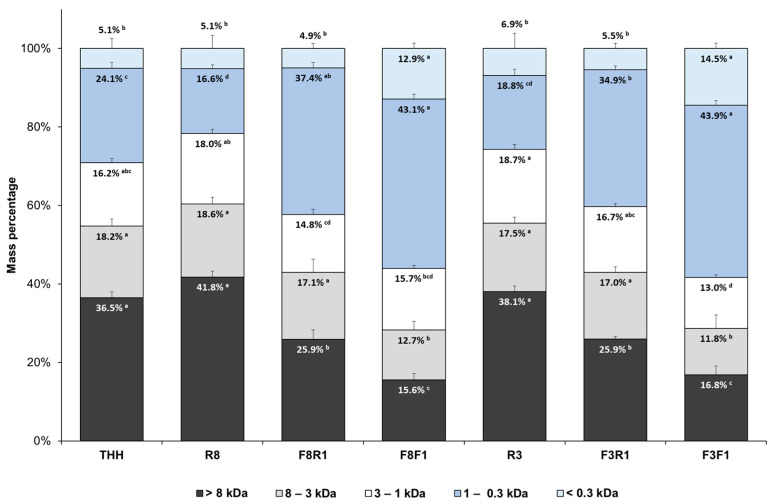
Molecular weight distribution of the UF peptide fractions (percentage of curve area). All the data are expressed as mean ± standard deviation of triplicate measurements. Different superscript letters indicate significant differences (*p* < 0.05) among the UF streams. THH: tuna head hydrolysate. R8, R3: retentate fractions from the 8 and 3 kDa MWCO membranes, respectively. F8R1, F3R1: retentate streams obtained after filtration through 1 kDa of F8 and F3, respectively. F8F1, F3F1: permeate fractions obtained after filtration through 1 kDa of F8 and F3, respectively.

The addition of Flavourzyme did not significantly affect the overall DH; however, it promoted the release of free amino acids and low-molecular-weight peptides, predominantly dipeptides. This is supported by the molecular weight distribution of the crude hydrolysate (THH), shown in Figure 3, where peptides below 1 kDa accounted for 29.2 ± 2.4% *w*/*w* of the total area.

The sequential application of Alcalase followed by Flavourzyme exhibited a synergistic effect: Alcalase initially hydrolyzed the globular proteins into larger peptide fragments, which were subsequently cleaved at their N-terminal sites by the exopeptidases present in the Flavourzyme complex. This combined treatment is commonly reported in the literature to obtain bioactive short-chain peptides or high protein recovery from fish by-products such as tilapia scales [43], cod heads [44], or sturgeon cartilage [45], among others.

### 3.2. Fluid-Dynamic Study of the Membrane Filtration

#### 3.2.1. Water Permeability of the Ceramic Membranes

The water permeability of the three ceramic membranes tested (8, 3, and 1 kDa) was evaluated by linear regression of the observed values of water flux (Jw) against the applied transmembrane pressure (TMP). The intrinsic membrane resistance (R_M_) was calculated as the inverse of the slope of Jw against TMP, as illustrated in Figure S1. Table 1 summarizes the values of R_M_ (bar·m^2^·h/L) for the three ceramic membranes studied. In accordance with Hagen–Poiseuille’s law, membrane resistance to water flux increased as the molecular weight cut-off (MWCO) of the membranes decreased, varying from 1.31 × 10^−2^ to 2.12 × 10^−2^ bar·m^2^·h/L for 8 and 1 kDa MWCO, respectively.

#### 3.2.2. Time Evolution of the Permeate Flux During Batch Concentration

**Figure 4:**
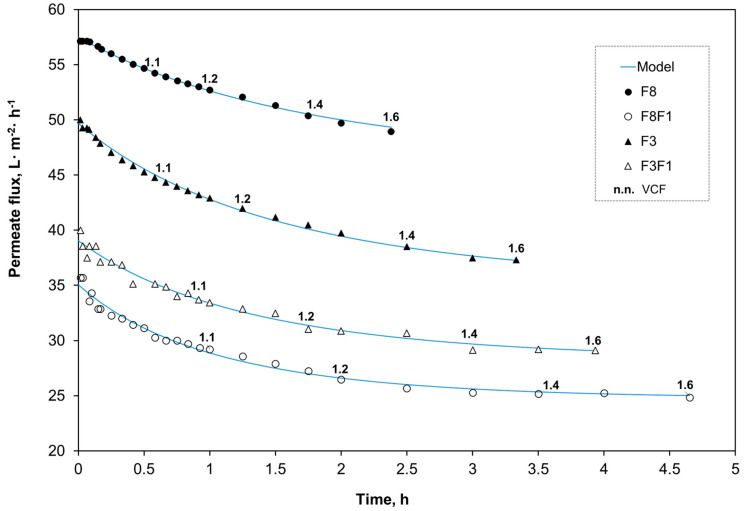
Evolution of the permeate flux (L·m^−2^ membrane·h^−1^) as a function of the operation time (h) and the volume concentration factor (VCF). The blue solid curves represent the permeate flux predicted by the dynamic resistance-in-series model (Equation (5)). F8, F3: permeate streams from the 8 and 3 kDa MWCO membranes, respectively. F8F1, F3F1: permeate streams obtained after filtration through 1 kDa of F8 and F3, respectively.

Figure 4 shows the evolution of the permeate flux during batch ultrafiltration, up to the final volume concentration factor (VCF) of 1.6. The total duration of the concentration process ranged from 2.4 h (8 kDa membrane fed with THH) to 4.7 h (1 kDa membrane processing F8). In general, the permeate flux declined markedly during the initial 60–90 min of concentration (corresponding to VCF 1.1–1.2), after which it gradually stabilized, reaching a near-steady state. This trend was more evident in the case of the 1 kDa MWCO membrane. Such behavior, widely reported in crossflow ultrafiltration, is attributed to the formation of a dynamic fouling layer on the membrane surface. The growth of this layer is counteracted by the shear forces of the retentate stream, leading to a steady value of permeate flux J_ꝏ_ [46].

The experimental permeate flux values were fitted to the dynamic resistance-in-series model described by Equation (4). The model parameters—initial flux (J_0_), steady-state flux (Jꝏ), and the fouling rate constant (k)—were estimated by nonlinear regression and are summarized in Table 1. The model showed a good fit to the experimental data, with coefficients of determination (R^2^) ranging from 0.9723 for the permeate F3F1 to 0.9977 for F8. The solid lines in Figure 3 represent the model predictions, showing good agreement with the observed data.

The permeation rate of peptide species increased with the membrane molecular weight cut-off (MWCO), as evidenced by J_0_ and J_ꝏ_ values. In the final filtration step using the 1 kDa membrane, a higher resistance was observed for stream F8 compared to F3. This behavior can be attributed to the MW distribution of F8, which contains a greater proportion of large peptides that may contribute to membrane fouling. This was confirmed by the higher estimated value of the fouling rate constant k (0.7227 h^−1^).

All membranes used in this study featured an active layer composed of titanium dioxide, which exhibits a point of zero charge (i.e., the pH at which the membrane surface has no net electrical charge) near pH 7 in aqueous solutions [47]. Under the operating conditions, this implies that the membrane surfaces carried a neutral to slightly negative net charge. Therefore, it can be concluded that the fouling rate constant (k) was primarily influenced by the membrane’s molecular weight cut-off (MWCO) and the concentration of peptides present in the feed streams.

#### 3.2.3. Cleaning Efficiency of the Alkaline Treatment

Following batch concentration, the fouled membranes underwent alkaline cleaning for 30 min at 50 °C, as previously described. The cleaning protocol concluded with a final rinse with water until the effluent reached neutral pH. The hydraulic resistance was then determined (Figure S1), calculating the cleaning efficiency of the alkaline treatment by Equation (5). Table 1 reports the hydraulic resistances of the clean (R_M_) and the fouled membranes after ultrafiltration (R_1_), as well as the efficiencies of the cleaning protocol.

A single alkaline stage fully restored the permeability of the 3 and 1 kDa membranes, achieving cleaning efficiencies above 99%. This high performance is attributed to the alkaline hydrolysis of proteins adsorbed on the membrane surface and within the pores, releasing soluble peptides that are removed during rinsing [48,49]. In contrast, the 8 kDa membrane showed only a 67% recovery after a single cleaning cycle, requiring a second treatment. In macroporous membranes, fouling is controlled by protein adsorption within pores rather than surface deposition or cake formation. Alkaline agents are generally less effective at removing internal foulants than those on the surface, a phenomenon also observed in whey protein filtration using MF ceramic membranes [50].

### 3.3. Evaluation of the Peptide Fractions as Natural Antioxidants for Aquaculture Diets

#### 3.3.1. Content of Soluble Protein and Amino Acid Composition of the Peptide Fractions

Table 2 presents the content of soluble protein of the membrane streams, which was determined by the BCA method. The crude hydrolysate presented a concentration of soluble protein of 9.35 ± 0.33 g·L^−1^. The different membrane fractions presented average levels of soluble protein ranging from 5.17 ± 0.14 g·L^−1^ (F3F1) to 10.73 ± 0.26 g·L^−1^ (R8). The retentates from the first membrane separation stage through 8 or 3 kDa presented higher contents of protein, which progressively declined throughout the fractionation.

Two membrane fractions, F3R1 and F3F1—retentate and permeate streams resulting from the sequential filtration of THH through 3 and 1 kDa membranes—were analyzed for their amino acid profile, expressed in mol percentage (Table 2). Overall, membrane purification did not significantly alter the amino acid profile, as all peptide fractions originated from the same enzymatic hydrolysate. The analytical method involved complete acid hydrolysis of the protein content, ensuring that the released amino acids were uniformly distributed between the permeate and retentate streams. Additionally, it should be noted that tryptophan (THR) and cysteine (CYS) were not determined by the analytical procedure due to their degradation after the acid digestion pretreatment.

Aquatic species require a well-balanced proportion of dietary amino acids for their development. These are substrates for protein synthesis and contribute to the regulation of several physiological processes of the farmed fish such as feed intake, oxygen and carbon dioxide transport, metabolism, cell signaling, or immune response [51]. Amino acids are categorized as essential (EAAs), conditionally essential, and non-essential (NEAAs) amino acids. The former cannot be synthesized by farmed animals and should be supplied by diet. In total, 10 amino acids are identified as essential for fish species (Table 2). According to FAO recommendations for farmed fish, they should account for 40–60% of the protein dietary intake [52]. As shown in Table 2, the protein fraction of both membrane streams presented a relevant proportion of EAAs, above 64% mol of crude protein. Both protein fractions presented a similar amino acid profile, characterized by high levels of arginine (18.2%), glycine (16.2–17.5%), leucine (8.2–9.1%), phenylalanine (8.0–8.7%), and valine (7.6–7.9%). The average lysine content of both membrane fractions was 3.14 ± 0.32% *w*/*w* of total protein, below dietary recommendations (5.5–6.5% *w*/*w* of protein content) [52]. This essential amino acid is considered a limiting factor in fish nutrition, and its actual content should be considered when including these protein fractions in aquafeeds.

#### 3.3.2. Molecular Weight Distribution of the Peptide Fractions

Fish protein hydrolysis is a mild technology that generates peptides with enhanced nutritional value and a range of bioactive properties, including antioxidant, antihypertensive, antimicrobial, and secretagogue activities, among others. However, the relationship between peptide structure and biological function is still not fully understood, as it is influenced by multiple factors such as molecular weight (MW) distribution, secondary structure of the bioactive peptides, amino acid sequence, hydrophobicity, net charge, and acid–base characteristics. Several studies highlight the role of the MW distribution of hydrolysates on their bioavailability and potential bioactivity [13,53,54]. In this context, membrane-based fractionation of fish protein hydrolysates (FPHs) allows the isolation of peptide fractions within specific MW ranges, which may present improved bioactivity with respect to the intact protein.

The MW distribution of the product streams R8, F8R1, F8F1, R3, F3R1, and F3F1 is presented in Figure 3. The SEC profiles were divided into five fractions: A (>8 kDa), B (3–8 kDa), C (1–3 kDa), D (0.3–1 kDa), and E (<0.3 kDa) (Figures S2 and S3). High-MW peptides above 3 kDa (fractions A+B) dominated the retentate streams R8 and R3, likely consisting of intact protein and large peptides generated by Alcalase. They underwent further hydrolysis by Flavourzyme, yielding short-chain peptides (mostly 2–3 residues) and free amino acids. The second UF stage through 1 kDa significantly increased the proportion of low-MW peptides, with fraction D being predominant in both the final retentate and permeate streams.

Peptide fractions recovered through membrane fractionation show promise as feed ingredients due to their high digestibility and potential bioactivity. Although the underlying mechanisms are not fully understood, several studies have reported that dietary peptide supplementation improves larval rearing outcomes, including higher survival rates, increased weight gain, and reduced skeletal abnormalities [55,56,57,58]. Kotzamanis et al. [59], for instance, recommend moderate levels of peptides in the range 500–2500 Da for optimal larval development. In this regard, the fractions C and D, representing 56–58% of the membrane fractions F8F1 and F3F1, could find application as ingredients in aquaculture formulations. Moreover, free amino acids have been reported to improve feed palatability and promote weight gain in juvenile and adult fish [23,60,61]. In this study, the amino acid content of the permeate streams F8F1 and F3F1 supports their potential use as attractant ingredients in aquafeeds.

#### 3.3.3. In Vitro Antioxidant Activities of the Peptide Fractions

Antioxidant supplementation is justified both to restrain lipid oxidation in aquafeeds themselves and to neutralize the reactive oxygen species (ROS) generated under fish farming conditions. The oxidation of lipid ingredients is a major concern for the fish feed industry, since it reduces the stability and shelf life of aquafeeds. Moreover, the conditions in aquaculture facilities—such as high stock density, alterations in environmental factors (e.g., pH, temperature, salinity, ammonia, heavy metals, and light exposure), pathogens, and other exogenous stressors—promote the release and accumulation of ROS (e.g., H_2_O_2_, HOCl, OH•, and O_2_−) in fish tissues. These are highly reactive molecules that can damage lipids, proteins, carbohydrates, or nucleic acids, altering their normal function [28,62].

The supplementation of aquafeeds with natural antioxidants, such as vitamins, carotenoids, polyphenols, or peptides, avoids the potential human risks related to the use of synthetic antioxidants (e.g., ethoxyquin, butylated hydroxytoluene, and tert-butyl hydroquinone) [62,63]. Unlike other natural antioxidants, marine antioxidant peptides have been less investigated. Although their structure–activity relationship has not been fully elucidated, most of the antioxidant peptides identified so far have between 3 and 20 amino acid residues and contain hydrophobic residues (e.g., Val, Trp, Phe, and Pro) within their sequence [28]. Their in vitro antioxidant activity is commonly assessed through radical scavenging assays using compounds such as 2,2- diphenyl-1-picrylhydrazyl (DPPH) or 2,2′-azino-bis(3-ethylbenzothiazoline-6-sulphonic acid) (ABTS). Another group of antioxidant assays evaluates the ability of peptides to chelate metal ions such as Fe^2+^ or Cu^+^, which catalyze lipid oxidation reactions [64].

**Figure 5:**
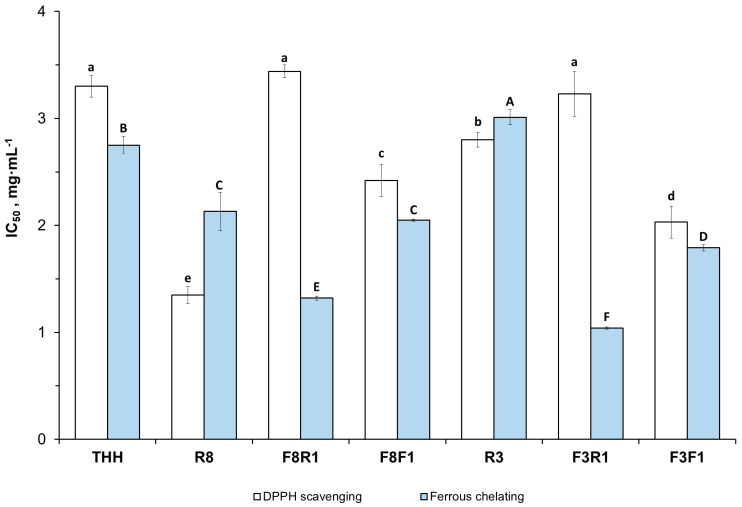
In vitro DPPH radical scavenging and ferrous chelating half-maximal inhibition (IC_50_, mg·mL^−1^) of the membrane UF fractions. Different superscript upper- (A, B, C, D, E, F) and lowercase letters (a, b, c, d, e) indicate significant statistical differences (*p* < 0.05) among membrane fractions for DPPH radical scavenging and ferrous chelating activities, respectively. THH: tuna head hydrolysate. R8, R3: retentate fractions from the 8 and 3 kDa MWCO membranes, respectively. F8R1, F3R1: retentate streams obtained after filtration through 1 kDa of F8 and F3, respectively. F8F1, F3F1: permeate fractions obtained after filtration through 1 kDa of F8 and F3, respectively.

In this study, two in vitro antioxidant activities, namely DPPH radical scavenging and Fe^2+^ chelating, were investigated in the peptide fractions obtained by sequential membrane processing. Figure 5 compares the average values of the half-maximal inhibitory concentration (IC_50_, mg·mL^−1^) of both bioactivities.

The results show significant differences (*p* < 0.05) among the peptide fractions, although no correlation was found between DPPH radical scavenging activity and MW profile. For instance, the retentate fractions F8R1 and F3R1 presented no significant differences with respect to the crude hydrolysate THH, although their content of short-chain peptides below 1 kDa was significantly higher (Figure 3). The retentate fraction R8 presented no significant differences in MW for THH except for the fraction D (0.3–1 kDa), which was significantly lower. Despite its similar profile, R8 presented the lowest IC_50_ value for in vitro DPPH radical inhibition (IC_50_ 1.35 ± 0.08 mg·mL^−1^), followed by F3F1 (2.03 ± 0.15 mg·mL^−1^). The latter is a permeate stream. Both fractions showed a significant improvement in DPPH radical inhibition with respect to the crude hydrolysate THH (IC_50_ 3.32 ± 0.10 mg·mL^−1^).

There is no common opinion in the scientific literature regarding the relationship between peptide MW and DPPH radical scavenging activity. Most of the radical scavenging peptides isolated so far from tuna by-products are short-chain peptides (<1 kDa) containing hydrophobic and aromatic amino acid residues (e.g., His, Pro, Tyr, and Val). This is related to their ability to donate hydrogen atoms/electrons to the radical [9,13,65] or enhance the solubilization of the active peptides into the lipid phase, where they interact with free radicals [8,66]. Similarly, Chi et al. [67] investigated the influence of amino acid and peptide compositions on the antioxidant activities of Skipjack tuna hydrolysates obtained with different commercial proteases. The authors reported that the hydrolysates showing strong radical scavenging activities presented a high proportion of low-MW peptides (between 3 and 6 amino acid residues, ~500 kDa) with relevant content of hydrophobic and aromatic amino acids. On the contrary, Krasae et al. [68] and Sisa [10] found that large peptides >10 kDa displayed high in vitro DPPH radical inhibitory activity in tuna viscera autolysates and yellowfin tuna tails, respectively. This was attributed to either the higher occurrence of hydrophobic residues in large peptides [68] or their ability to retain the secondary structure [10].

Regardless of chain length, peptide hydrophobicity is generally accepted to enhance radical scavenging activity. As shown in Table 2, both F3R1 and F3F1 streams presented a similar content of hydrophobic amino acids (>60% mol.) although the proportion of short-chain peptides below 1 kDa was significantly higher in F3F1. This fraction presented the second lowest IC_50_ for DPPH radical scavenging activity.

As a general trend, our results indicate that the removal of large peptides by ultrafiltration through 8 and 3 kDa MWCO membranes had a positive impact on the in vitro Fe^2+^ chelating activity. The retentate streams F3R1 and F8R1 exhibited the highest chelating potency, with IC_50_ values of 1.04 ± 0.01 mg·mL^−1^ and 1.32 ± 0.02 mg·mL^−1^, respectively. Both fractions presented similar MW profiles, where the peptide fraction C+ D (0.3–3 kDa) accounted for 52.1 ± 3.6% and 51.6 ± 1.7% of the MW distribution, respectively. The permeate streams obtained from the 1 kDa filtration exhibited slightly higher IC_50_ values, though still improved in comparison to the crude hydrolysate (IC_50_ 2.75 ± 0.08 mg·mL^−1^). Short-chain peptides are more soluble, exhibiting higher mobility to interact with metal ions. The presence of histidine, cysteine, or negatively charged residues such as glutamic or aspartic acid is reported to enhance metal chelation [8,66,69], although non-charged polar groups such as hydroxyl, present in serine and threonine residues, can actively participate in metal coordination [70]. Additionally, other studies highlight the role of aromatic and hydrophobic residues (e.g., Leu and Phe) in stabilizing peptide–metal complexes [71]. The improved metal chelation activity displayed by fractions F3R1 and F3F1 could be explained by their content of low-MW peptides (<1 kDa) and amino acid composition.

It should be noted that in vitro assays serve as a preliminary screening step to assess whether protein-derived or other natural molecules have the potential to exert antioxidant effects. In vivo assays using cell lines or aquatic models (e.g., zebrafish larvae and farmed fish species) are required to accurately evaluate how these bioactive molecules mitigate oxidative stress under physiological conditions.

## 4. Conclusions

Skipjack tuna heads were sequentially hydrolyzed with Alcalase (4 h) and Flavourzyme (1 h), reaching a final DH of 18.5 ± 0.9%. The resulting crude hydrolysate was fractionated by ceramic membranes with MWCOs of 8, 3, and 1 kDa to obtain peptide fractions, which were characterized for their protein content, MW distribution, and in vitro antioxidant activities. This approach allowed the recovery of peptide fractions (e.g., the permeate streams F8F1 and F3F1) containing more than 50% *w*/*w* short-chain peptides (0.3–3 kDa) and 12–15% *w*/*w* free amino acids. Besides their specific peptide composition, these fractions also exhibited a high proportion of essential amino acids (>64% mol), supporting their use as functional dietary ingredients in aquafeeds.

In vitro assays revealed that certain membrane fractions showed enhanced DPPH scavenging and metal chelation activities compared to the crude hydrolysate, which may be associated with their MW profile and hydrophobic amino acid content. Overall, the integrated process proposed in this study allows the valorization of Skipjack tuna head by-products into functional peptide fractions with a desirable MW distribution and amino acid composition and potential antioxidant activities. Future research should focus on assessing the effects of these fractions on feed palatability, digestibility, and their capacity to mitigate oxidative stress through in vivo zootechnical trials.

## Figures and Tables

**Table 1:** Hydraulic resistances of the ceramic membranes before (clean, RM) and after batch concentration (fouled, R1). Parameters of the resistance-in-series model: initial permeate flux (J_0_), steady permeate flux (J_∞_) and fouling rate constant (k). Percentage cleaning efficiency of the alkaline treatment. Note that the 8 kDa MWCO membrane required a second cleaning stage to recover its full permeability. F8, F3: permeate streams from the 8 and 3 kDa MWCO membranes, respectively. F8F1, F3F1: Permeate streams obtained after filtration through 1 kDa of F8 and F3, respectively.

Membrane MWCO	Membrane Resistances		Resistance-in-Series Model, Equation (4)				% Cleaning Efficiency, Equation (5)	
	Initial, R_M_ Bar·m^2^·h·L^−1^	Fouled, R_1_ Bar·m^2^·h·L^−1^	Permeate Stream	J_0_ L·m^−2^·h^−1^	J_∞_ L·m^−2^·h^−1^	k h^−1^	1st Stage	2nd Stage
8 kDa	1.31 × 10^−2^	2.50 × 10^−2^	F8	57.49	46.28	0.4794	66.87	23.13
3 kDa	1.98 × 10^−2^	3.10 × 10^−2^	F3	49.66	35.16	0.4933	99.79	-
1 kDa fed F8	2.12 × 10^−2^	3.75 × 10^−2^	F8F1	35.04	24.75	0.7227	99.90	-
1 kDa fed F3	2.12 × 10^−2^	4.02 × 10^−2^	F3F1	39.06	28.28	0.5892	99.24	-

**Table 2:** Concentration (g·L^−1^) of soluble protein of the crude hydrolysate and the membrane fractions. Amino acid composition (mol %) of the membrane streams F3R1 and F3F1. THH: tuna head hydrolysate. R8, R3: retentate fractions from the 8 and 3 kDa MWCO membranes, respectively. F8R1, F3R1: retentate streams obtained after filtration through 1 kDa of F8 and F3, respectively. F8F1, F3F1: permeate fractions obtained after filtration through 1 kDa of F8 and F3, respectively.

Content of Soluble Protein		Amino Acid Composition of F3R1 and F3F1		
Stream	Concentration, g·L^−1^	Amino Acid	Mol Percentage	
			F3R1	F3F1
THH	9.35 ± 0.33	ALA	7.41 ± 0.15	6.73 ± 0.22
R8	10.73 ± 0.26	ARG	18.16 ± 0.96	18.29 ± 0.67
F8R1	6.36 ± 0.49	ASN + ASP	0.05 ± 0.00	0.05 ± 0.00
F8F1	4.92 ± 0.29	GLN + GLU	1.50 ± 0.15	1.49 ± 0.21
R3	10.29 ± 0.29	GLY	16.19 ± 0.67	17.46 ± 0.41
F3R1	5.50 ± 0.56	HIS	3.86 ± 0.13 *	4.62 ± 0.19
F3F1	5.17 ± 0.14	ILE	4.93 ± 0.16	5.17 ± 0.13
		LEU	8.22 ± 0.19 *	9.06 ± 0.13
		LYS	2.85 ± 0.12	2.86 ± 0.17
		MET	4.71 ± 0.19	3.97 ± 0.21
		PHE	8.65 ± 0.12 *	8.04 ± 0.07
		PRO	1.56 ± 0.07 *	0.68 ± 0.12
		SER	3.92 ± 0.25	3.39 ± 0.23
		THR	4.51 ± 0.48	4.52 ± 0.78
		TYR	5.64 ± 0.24	6.04 ± 0.14
		VAL	7.85 ± 0.41	7.61 ± 0.25
Total Amino Acids ^1^			100 ± 1.44	100 ± 1.27
Total Essential Amino Acids ^2^			64.74 ± 1.21	64.14 ± 1.13
Total Hydrophobic Amino Acids ^3^			63.16 ± 0.90	64.76 ± 0.63

^1^ Tryptophan (TRP) and cysteine (CYS) were not determined by the amino acid analysis. ^2^ Essential Amino Acids: ARG, HIS, ILE, LEU, LYS, MET, PHE, THR, TRP (not detected), and VAL. ^3^ Hydrophobic Amino Acids: ALA, GLY, ILE, LEU, MET, PHE, PRO, TRP (not detected), TYR, and VAL. * Significant differences (*p* < 0.005) between F3R1 and F3F1.

## Data Availability

The original contributions presented in this study are included in the article. Further inquiries can be directed to the corresponding author.

## Supplementary Materials

The following supporting information can be downloaded at: https://www.mdpi.com/article/10.3390/antiox14070770/s1, Figure S1: Water flux (L·m^−2^) against transmembrane pressure (bar) for the ceramic ultrafiltration membranes at different operation stages: initial (before ultrafiltration, blue straight line), fouled (after ultrafiltration until attaining a volume concentration factor of 1.6) and after alkaline cleaning treatment. (a) 8 kDa MWCO membrane fed tuna head hydrolysates (THH); (b) 3 kDa MWCO membrane fed tuna head hydrolyates; (c) 1 kDa MWCO membrane fed the permeate stream from the 8 kDa membrane (F8); (d) 1 kDa MWCO membrane fed the permeate stream from the 3 kDa membrane (F3); Figure S2: Size Exclusion Chromatograms for the crude tuna head hydrolysate (THH, top left); the retentate obtained after filtration of THH through 8 kDa (R8, top right), the permeate obtained after successive filtration of THH through 8 and 1 kDa (F8F1, bottom left), and the retentate obtained after successive filtration of THH through 8 and 1 kDa (F8R1, bottom right). Actual absorbance values (mAU) at 285 nm were normalized so that the total area below curve is unitary; Figure S3: Size Exclusion Chromatograms for the retentate obtained after filtration of THH through 3 kDa (R3, top left); the retentate obtained after successsive filtration of THH through 3 and 1 kDa (F3R1, top right), and the permeate obtained after successive filtration of THH through 3 and 1 kDa (F3F1, bottom right). Actual absorbance values (mAU) at 285 nm were normalized so that the total area below curve is unitary.

## References

[1] FAO (2024). The State of World Fisheries and Aquaculture 2024.

[2] Ghalamara S., Brazinha C., Silva S., Pintado M. (2024). Exploring Fish Processing By-Products as an Alternative Source of Bioactive Peptides: A Review on Extraction and Food Applications. Curr. Food Sci. Technol. Rep..

[3] The Spanish Canned Seafood Industry in 2024: An Overview of the Sector|Gaictech®. https://www.gaictech.com/en/blog/the-spanish-canned-seafood-industry-in-2024-an-overview-of-the-sector/.

[4] Ramakrishnan S.R., Jeong C.-R., Park J.-W., Cho S.-S., Kim S.-J. (2023). A Review on the Processing of Functional Proteins or Peptides Derived from Fish By-Products and Their Industrial Applications. Heliyon.

[5] Zhang Y., Sun Q., Liu S., Wei S., Xia Q., Ji H., Deng C., Hao J. (2021). Extraction of Fish Oil from Fish Heads Using Ultra-High Pressure Pre-Treatment Prior to Enzymatic Hydrolysis. Innov. Food Sci. Emerg. Technol..

[6] Ma C.-C., Wang X.-C., Tao N.-P. (2021). Hydroxyapatite from the Skull of Tuna (*Thunnus obesus*) Head Combined with Chitosan to Restore Locomotive Function After Spinal Cord Injury. Front. Nutr..

[7] López-Álvarez M., Souto-Montero P., Durán S., Pérez-Davila S., Vázquez J.A., González P., Serra J. (2024). Valuable Ca/P Sources Obtained from Tuna Species’ By-Products Derived from Industrial Processing: Physicochemical and Features of Skeleton Fractions. Recycling.

[8] Wang Y.M., Li X.Y., Wang J., He Y., Chi C.F., Wang B. (2022). Antioxidant Peptides from Protein Hydrolysate of Skipjack Tuna Milt: Purification, Identification, and Cytoprotection on H_2_O_2_ Damaged Human Umbilical Vein Endothelial Cells. Process Biochem..

[9] Zhang S.-Y., Zhao Y.-Q., Wang Y.-M., Yang X.-R., Chi C.-F., Wang B. (2022). Gelatins and Antioxidant Peptides from Skipjack Tuna (*Katsuwonus pelamis*) Skins: Purification, Characterization, and Cytoprotection on Ultraviolet-A Injured Human Skin Fibroblasts. Food Biosci..

[10] Sisa A., Martínez-Álvarez O., Gómez-Estaca J., Mosquera M. (2024). Valorization of Yellowfin Tuna Tails: From Proteolytic Enzyme Production to Gelatin and Antioxidant Hydrolysate Extraction. Foods.

[11] Wardani D.W., Ningrum A., Manikharda, Vanidia N., Munawaroh H.S.H., Susanto E., Show P.L. (2023). In Silico and in Vitro Assessment of Yellowfin Tuna Skin (*Thunnus albacares*) Hydrolysate Antioxidation Effect. Food Hydrocoll. Health.

[12] Qiao Q.Q., Luo Q.B., Suo S.K., Zhao Y.Q., Chi C.F., Wang B. (2022). Preparation, Characterization, and Cytoprotective Effects on HUVECs of Fourteen Novel Angiotensin-I-Converting Enzyme Inhibitory Peptides From Protein Hydrolysate of Tuna Processing By-Products. Front. Nutr..

[13] Pezeshk S., Ojagh S.M., Rezaei M., Shabanpour B. (2019). Fractionation of Protein Hydrolysates of Fish Waste Using Membrane Ultrafiltration: Investigation of Antibacterial and Antioxidant Activities. Probiotics Antimicrob. Proteins.

[14] Prasetyo D.Y.B., Agustini T.W., Anjani G., Riyadi P.H. (2025). Evaluation of Immunomodulatory Properties of Fish-Protein Hydrolysate from Skipjack Tuna by-Products (*Katsuwonus pelamis*, Linnaeus 1758) in Streptozotocin-Nicotinamide-Induced Diabetic Rats. World’s Vet. J..

[15] Aquaculture in Spain 2024. https://apromar.es/wp-content/uploads/2025/03/Informe2024_ENG_v2.pdf.

[16] Hamre K., Yúfera M., Rønnestad I., Boglione C., Conceição L.E.C., Izquierdo M. (2013). Fish Larval Nutrition and Feed Formulation: Knowledge Gaps and Bottlenecks for Advances in Larval Rearing. Rev. Aquac..

[17] Ahmed N., Thompson S., Glaser M. (2019). Global Aquaculture Productivity, Environmental Sustainability, and Climate Change Adaptability. Environ. Manag..

[18] Coppola D., Lauritano C., Palma Esposito F., Riccio G., Rizzo C., de Pascale D. (2021). Fish Waste: From Problem to Valuable Resource. Mar. Drugs.

[19] Chaklader M.R., Siddik M.A.B., Fotedar R. (2020). Total Replacement of Fishmeal with Poultry Byproduct Meal Affected the Growth, Muscle Quality, Histological Structure, Antioxidant Capacity and Immune Response of Juvenile Barramundi, Lates Calcarifer. PLoS ONE.

[20] Chaklader M.R., Howieson J., Siddik M.A.B., Foysal M.J., Fotedar R. (2021). Supplementation of Tuna Hydrolysate and Insect Larvae Improves Fishmeal Replacement Efficacy of Poultry By-Product in Lates Calcarifer (Bloch, 1790) Juveniles. Sci. Rep..

[21] Siddik M.A.B., Howieson J., Fotedar R., Partridge G.J. (2021). Enzymatic Fish Protein Hydrolysates in Finfish Aquaculture: A Review. Rev. Aquac..

[22] Rashidian G., Abedian Kenari A., Nikkhah M. (2021). Dietary Effects of a Low-Molecular Weight Fraction (<10 KDa) from Shrimp Waste Hydrolysate on Growth Performance and Immunity of Rainbow Trout (Oncorhynchus Mykiss): Employing Nanodelivery Systems. Fish. Shellfish. Immunol..

[23] Barroso F.G., Rodiles A., Vizcaino A.J., Martínez T.F., Alarcón F.J. (2013). Evaluation of Feed Attractants in Juvenile Senegalese Sole, Solea Senegalensis. J. World Aquac. Soc..

[24] Chotikachinda R., Tantikitti C., Benjakul S., Rustad T., Kumarnsit E. (2013). Production of Protein Hydrolysates from Skipjack Tuna (*Katsuwonus pelamis*) Viscera as Feeding Attractants for Asian Seabass (*Lates calcarifer*). Aquac. Nutr..

[25] Valero Y., Saraiva-Fraga M., Costas B., Guardiola F.A. (2020). Antimicrobial Peptides from Fish: Beyond the Fight against Pathogens. Rev. Aquac..

[26] Kang H.K., Lee H.H., Seo C.H., Park Y. (2019). Antimicrobial and Immunomodulatory Properties and Applications of Marine-Derived Proteins and Peptides. Mar. Drugs.

[27] Kemp D.C., Kwon J.Y. (2021). Fish and Shellfish-Derived Anti-Inflammatory Protein Products: Properties and Mechanisms. Molecules.

[28] Aklakur M. (2018). Natural Antioxidants from Sea: A Potential Industrial Perspective in Aquafeed Formulation. Rev. Aquac..

[29] Chorhirankul N., Janssen A.E.M., Boom R.M. (2024). UF Fractionation of Fish Protein Hydrolysate. Sep. Purif. Technol..

[30] Alavi F., Ciftci O.N. (2023). Purification and Fractionation of Bioactive Peptides through Membrane Filtration: A Critical and Application Review. Trends Food Sci. Technol..

[31] Association of Official Analytical Chemists (2012). Official Methods of Analysis of the AOAC.

[32] Saadaoui H., Espejo-Carpio F.J., Guadix E.M., Amar R.B., Pérez-Gálvez R. (2019). Bi-Objective Optimization of Tuna Protein Hydrolysis to Produce Aquaculture Feed Ingredients. Food Bioprod. Process..

[33] Adler-Nissen J. (1986). Enzymic Hydrolysis of Food Proteins.

[34] Steinhardt H., Beychok S., Neurath H. (1964). Interaction of Proteins with Hydrogen Ions and Other Small Ions and Molecules. The Proteins.

[35] Suki A., Fane A.G., Fell C.J.D. (1984). Flux Decline in Protein Ultrafiltration. J. Memb. Sci..

[36] Pérez-Gálvez R., Guadix E.M., Bergé J.-P., Guadix A. (2011). Operation and Cleaning of Ceramic Membranes for the Filtration of Fish Press Liquor. J. Memb. Sci..

[37] Pérez-Gálvez R., Guadix E.M., Bergé J.-P., Guadix A. (2013). Processing Fish Press Waters Using Metallic and Ceramic Filtration. J. Chem. Technol. Biotechnol..

[38] Smith P.K., Krohn R.I., Hermanson G.T., Mallia A.K., Gartner F.H., Provenzano M.D., Fujimoto E.K., Goeke N.M., Olson B.J., Klenk D.C. (1985). Measurement of Protein Using Bicinchoninic Acid. Anal. Biochem..

[39] Picot L., Ravallec R., Martine F.P., Vandanjon L., Jaouen P., Chaplain-Derouiniot M., Guérard F., Chabeaud A., Legal Y., Alvarez O.M. (2010). Impact of Ultrafiltration and Nanofiltration of an Industrial Fish Protein Hydrolysate on Its Bioactive Properties. J. Sci. Food Agric..

[40] Decker E.A., Welch B. (1990). Role of Ferritin as a Lipid Oxidation Catalyst in Muscle Food. J. Agric. Food Chem..

[41] Hou Y., Wu Z., Dai Z., Wang G., Wu G. (2017). Protein Hydrolysates in Animal Nutrition: Industrial Production, Bioactive Peptides, and Functional Significance. J. Anim. Sci. Biotechnol..

[42] Merz M., Eisele T., Berends P., Appel D., Rabe S., Blank I., Stressler T., Fischer L. (2015). Flavourzyme, an Enzyme Preparation with Industrial Relevance: Automated Nine-Step Purification and Partial Characterization of Eight Enzymes. J. Agric. Food Chem..

[43] Sierra-Lopera L.M., Zapata-Montoya J.E. (2021). Optimization of Enzymatic Hydrolysis of Red Tilapia Scales (*Oreochromis* sp.) to Obtain Bioactive Peptides. Biotechnol. Rep..

[44] Remme J., Tveit G.M., Toldnes B., Slizyte R., Carvajal A.K. (2022). Production of Protein Hydrolysates from Cod (Gadus Morhua) Heads: Lab and Pilot Scale Studies. J. Aquat. Food Prod. Technol..

[45] Lin M.-Z., Chen B.-H. (2024). An Improved Production Method of Bioactive Peptides from Sturgeon Fish Cartilage. Food Bioproc. Tech..

[46] Corbatón-Báguena M.-J., Álvarez-Blanco S., Vincent-Vela M.-C. (2015). Fouling Mechanisms of Ultrafiltration Membranes Fouled with Whey Model Solutions. Desalination.

[47] Dutournié P., Limousy L., Anquetil J., Déon S. (2017). Modification of the Selectivity Properties of Tubular Ceramic Membranes after Alkaline Treatment. Membranes.

[48] Li K., Li S., Huang T., Dong C., Li J., Zhao B., Zhang S. (2019). Chemical Cleaning of Ultrafiltration Membrane Fouled by Humic Substances: Comparison between Hydrogen Peroxide and Sodium Hypochlorite. Int. J. Environ. Res. Public Health.

[49] Krishnan S., Nasrullah M., Kamyab H., Suzana N., Ab Munaim M.S., Ab Wahid Z., Ali I.H., Salehi R., Chaiprapat S. (2022). Fouling Characteristics and Cleaning Approach of Ultrafiltration Membrane during Xylose Reductase Separation. Bioprocess Biosyst. Eng..

[50] Mourouzidis-Mourouzis S.A., Karabelas A.J. (2008). Whey Protein Fouling of Large Pore-Size Ceramic Microfiltration Membranes at Small Cross-Flow Velocity. J. Memb. Sci..

[51] Xing S., Liang X., Zhang X., Oliva-Teles A., Peres H., Li M., Wang H., Mai K., Kaushik S.J., Xue M. (2024). Essential Amino Acid Requirements of Fish and Crustaceans, a Meta-Analysis. Rev. Aquac..

[52] FAO Nutritional Requirements. https://www.fao.org/fishery/affris/species-profiles/european-seabass/nutritional-requirements/en/.

[53] Karami Z., Akbari-adergani B. (2019). Bioactive Food Derived Peptides: A Review on Correlation between Structure of Bioactive Peptides and Their Functional Properties. J. Food Sci. Technol..

[54] Vázquez J.A., Comesaña S., Soengas J.L., Pérez M., Bermúdez R., Rotllant J., Valcarcel J. (2024). Optimal and Sustainable Production of Tailored Fish Protein Hydrolysates from Tuna Canning Wastes and Discarded Blue Whiting: Effect of Protein Molecular Weight on Chemical and Bioactive Properties. Sci. Total Environ..

[55] Cai Z., Li W., Mai K., Xu W., Zhang Y., Ai Q. (2015). Effects of Dietary Size-Fractionated Fish Hydrolysates on Growth, Activities of Digestive Enzymes and Aminotransferases and Expression of Some Protein Metabolism Related Genes in Large Yellow Croaker (*Larimichthys crocea*) Larvae. Aquaculture.

[56] Khosravi S., Bui H.T.D., Herault M., Fournier V., Kim K.-D., Lee B.-J., Kim K.-W., Lee K.-J. (2018). Supplementation of Protein Hydrolysates to a Low-Fishmeal Diet Improves Growth and Health Status of Juvenile Olive Flounder, Paralichthys Olivaceus. J. World Aquac. Soc..

[57] Ovissipour M., Kenari A.A., Nazari R., Motamedzadegan A., Rasco B. (2014). Tuna Viscera Protein Hydrolysate: Nutritive and Disease Resistance Properties for Persian Sturgeon (*Acipenser persicus* L.) Larvae. Aquac. Res..

[58] Printzi A., Jodet S., Fournier V., Collet S., Madec L., Simon V., Zambonino-Infante J.-L., Koumoundouros G., Mazurais D. (2024). Effect of Early Peptide Diets on European Sea Bass (*Dicentrarchus labrax*) Skeletal Development. Aquaculture.

[59] Kotzamanis Y.P., Gisbert E., Gatesoupe F.J., Infante J.Z., Cahu C. (2007). Effects of Different Dietary Levels of Fish Protein Hydrolysates on Growth, Digestive Enzymes, Gut Microbiota, and Resistance to Vibrio Anguillarum in European Sea Bass (*Dicentrarchus labrax*) Larvae. Comp. Biochem. Physiol.—A Mol. Integr. Physiol..

[60] Morais S. (2017). The Physiology of Taste in Fish: Potential Implications for Feeding Stimulation and Gut Chemical Sensing. Rev. Fish. Sci. Aquac..

[61] Li X., Fang T., Wang J., Wang Z., Guan D., Sun H., Yun X., Zhou J. (2022). The Efficiency of Adding Amino Acid Mixtures to a Diet Free of Fishmeal and Soybean Meal as an Attractant in Yellow River Carp (*Cyprinus carpio* Var.). Aquac. Rep..

[62] Hu X., Ma W., Zhang D., Tian Z., Yang Y., Huang Y., Hong Y. (2025). Application of Natural Antioxidants as Feed Additives in Aquaculture: A Review. Biology.

[63] Lundebyea A.K., Hovea H., Mågea A., Bohneb V.J.B., Hamrea K. (2010). Levels of Synthetic Antioxidants (Ethoxyquin, Butylated Hydroxytoluene and Butylated Hydroxyanisole) in Fish Feed and Commercially Farmed Fish. Food Addit. Contam. Part A Chem. Anal. Control. Expo. Risk Assess..

[64] López-García G., Dublan-García O., Arizmendi-Cotero D., Gomez-Olivan L.M. (2022). Antioxidant and Antimicrobial Peptides Derived from Food Proteins. Molecules.

[65] Unnikrishnan P., Kizhakkethil B.P., George J.C., Abubacker Z.A., Ninan G., Chandragiri Nagarajarao R. (2021). Antioxidant Peptides from Dark Meat of Yellowfin Tuna (*Thunnus albacares*): Process Optimization and Characterization. Waste Biomass Valorization.

[66] Wang J., Wang Y.M., Li L.Y., Chi C.F., Wang B. (2022). Twelve Antioxidant Peptides from Protein Hydrolysate of Skipjack Tuna (*Katsuwonus pelamis*) Roe Prepared by Flavourzyme: Purification, Sequence Identification, and Activity Evaluation. Front. Nutr..

[67] Chi C.-F., Hu F.-Y., Wang B., Li Z.-R., Luo H.-Y. (2015). Influence of Amino Acid Compositions and Peptide Profiles on Antioxidant Capacities of Two Protein Hydrolysates from Skipjack Tuna (*Katsuwonus pelamis*) Dark Muscle. Mar. Drugs.

[68] Krasae K., Worawattanamateekul W., Hinsui J. (2023). Effects of Peptide Fractions and Amino Acids on Antioxidant Properties of Autolyzed Tuna Viscera Protein Hydrolysate. Food Res..

[69] Lu W.-T., Dong C.-M. (2022). Research Progress of Metal Chelating Peptides. Food Health.

[70] Gulcin İ., Alwasel S.H. (2022). Metal Ions, Metal Chelators and Metal Chelating Assay as Antioxidant Method. Processes.

[71] Sun X., Sarteshnizi R.A., Boachie R.T., Okagu O.D., Abioye R.O., Neves R.P., Ohanenye I.C., Udenigwe C.C. (2020). Peptide–Mineral Complexes: Understanding Their Chemical Interactions, Bioavailability, and Potential Application in Mitigating Micronutrient Deficiency. Foods.

