# Supplementary material for: Processing of Tuna Head By-Products into Antioxidant Peptide Ingredients for Aquaculture Feeds
Source: Antioxidants (Basel). 2025 Jun 23;14(7):770. doi: 10.3390/antiox14070770 (PMC12291633; doi:10.3390/antiox14070770)

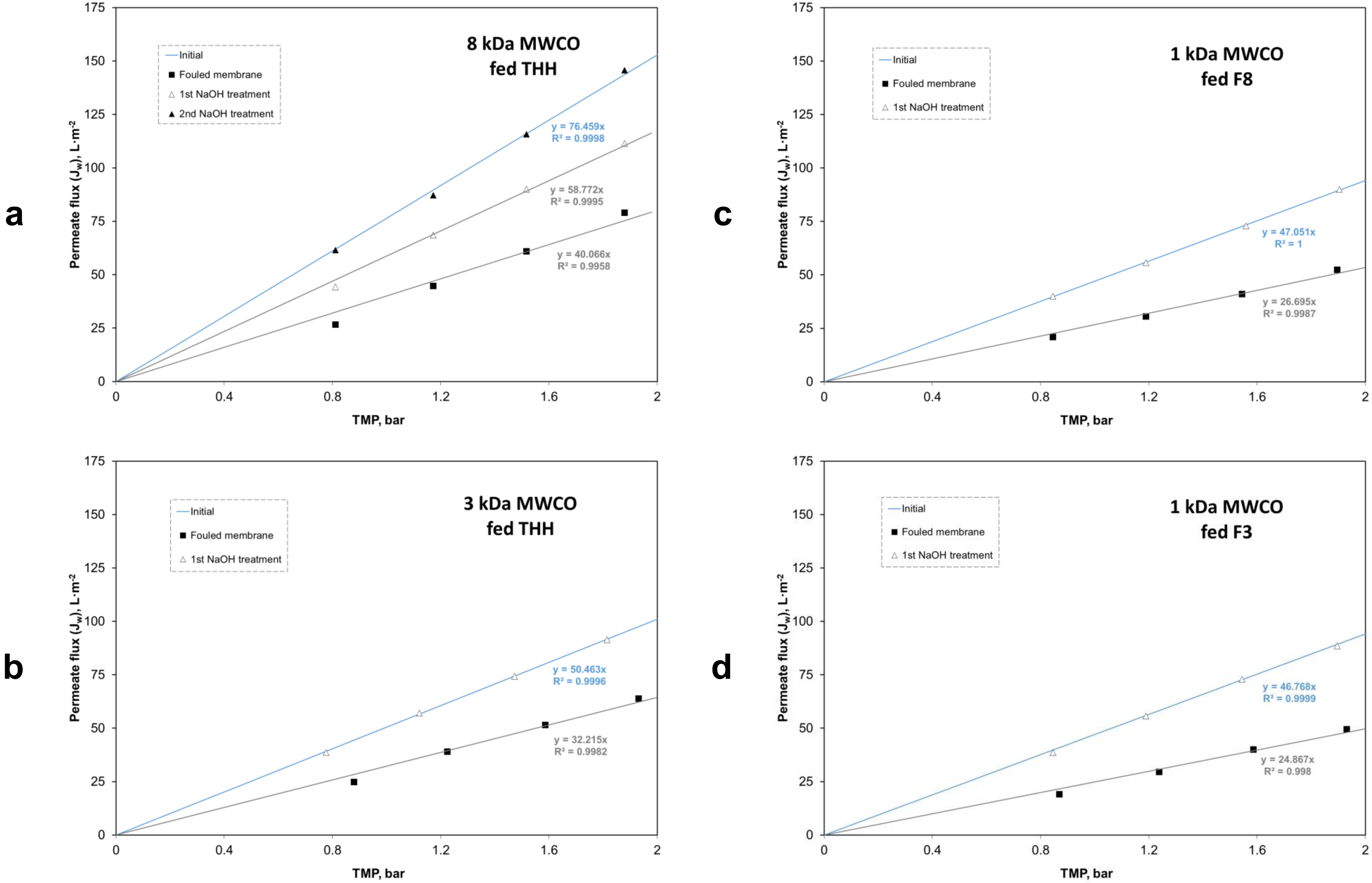


**Figure S1**. Water flux (L·m$^{-2}$) against transmembrane pressure (bar) for the ceramic ultrafiltration membranes at different operation stages: initial (before ultrafiltration, blue straight line), fouled (after ultrafiltration until attaining a volume concentration factor of 1.6) and after alkaline cleaning treatment. (a) 8 kDa MWCO membrane fed tuna head hydrolysates (THH); (b) 3 kDa MWCO membrane fed tuna head hydrolyates; (c) 1 kDa MWCO membrane fed the permeate stream from the 8 kDa membrane (F8); (d) 1 kDa MWCO membrane fed the permeate stream from the 3 kDa membrane (F3).

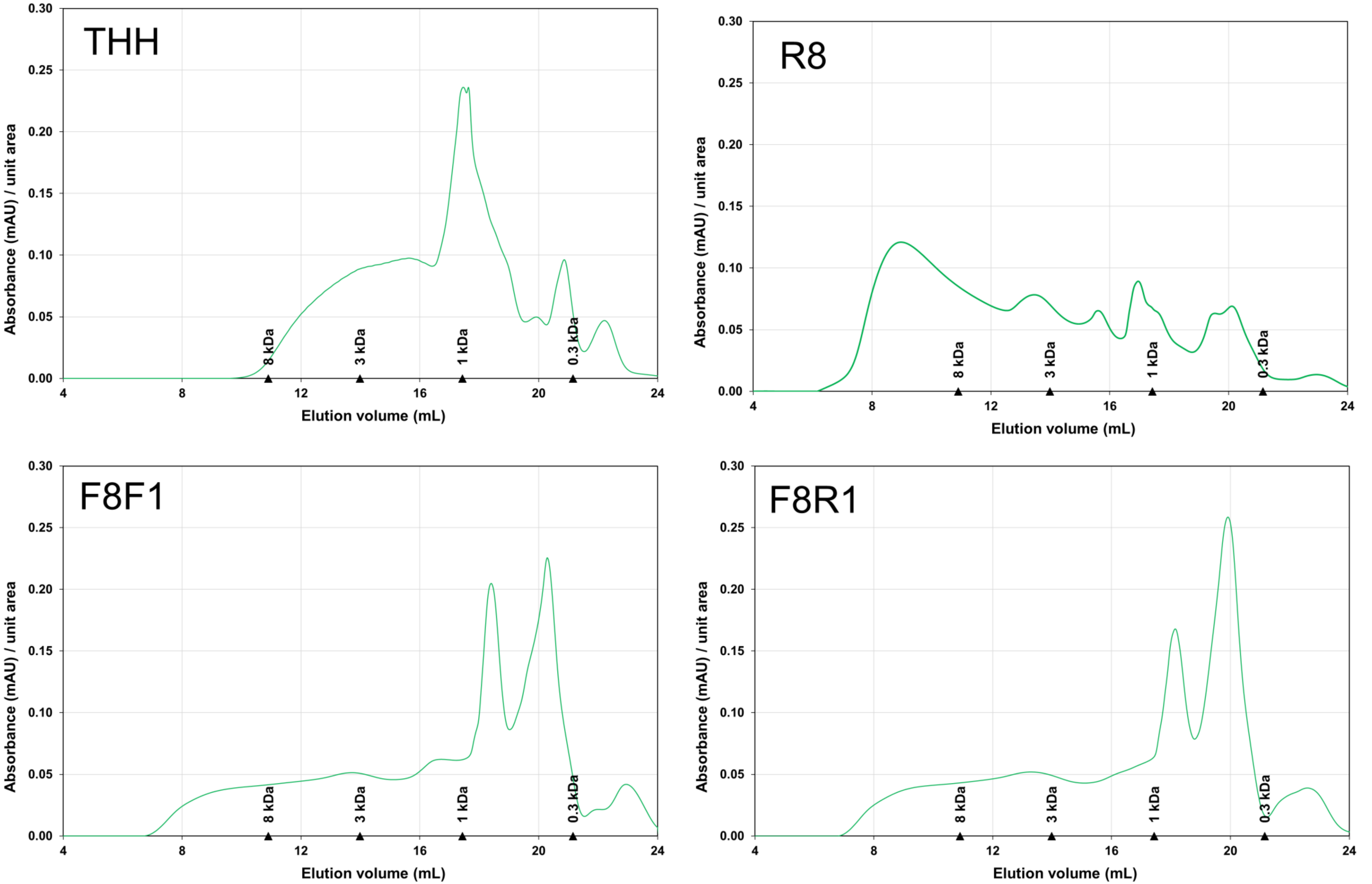


**Figure S2**. Size Exclusion Chromatograms for the crude tuna head hydrolysate (THH, top left); the retentate obtained after filtration of THH through 8 kDa (R8, top right), the permeate obtained after successive filtration of THH through 8 and 1 kDa (F8F1, bottom left), and the retentate obtained after successive filtration of THH through 8 and 1 kDa (F8R1, bottom right). Actual absorbance values (mAU) at 285 nm were normalized so that the total area below curve is unitary.

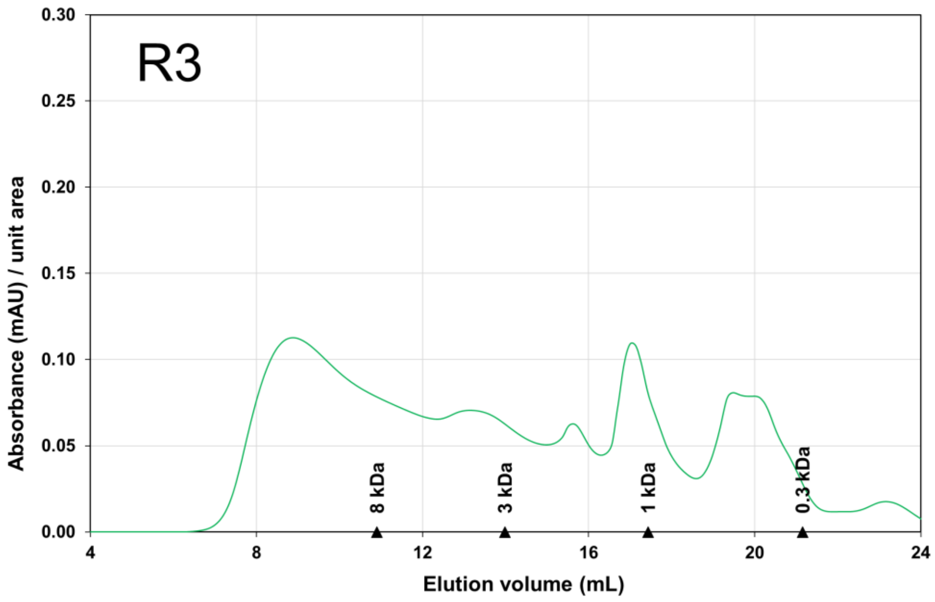


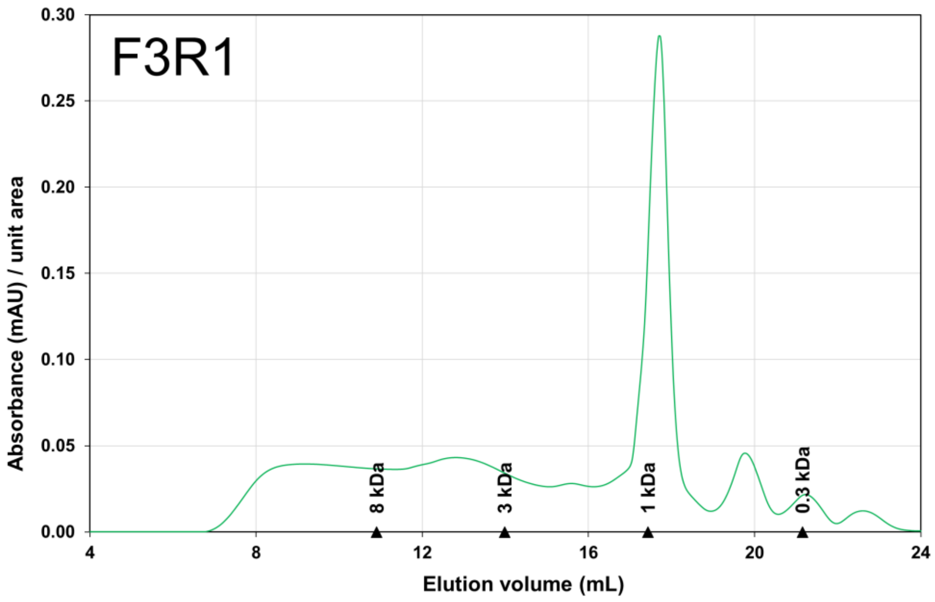


**Figure S3**. Size Exclusion Chromatograms for the retentate obtained after filtration of THH through 3 kDa (R3, top left); the retentate obtained after successsive filtration of THH through 3 and 1 kDa (F3R1, top right), and the permeate obtained after successive filtration of THH through 3 and 1 kDa (F3F1, bottom right). Actual absorbance values (mAU) at 285 nm were normalized so that the total area below curve is unitary.

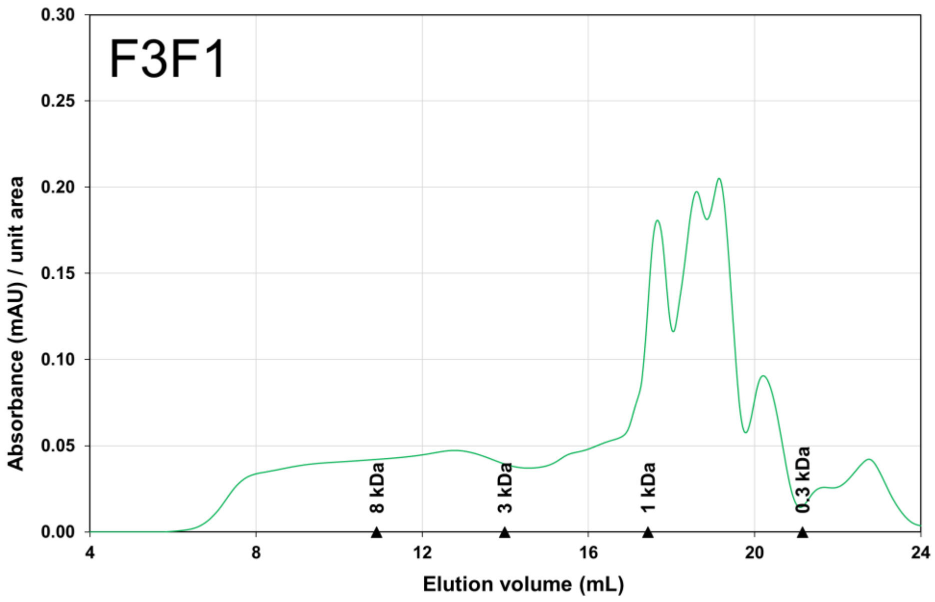

Supplement: Supplementary file 1 [file antioxidants-14-00770-s001.zip › antioxidants-3681044-supplementary.pdf]
